# Multiple blood gas variables predict AKI survival in an independent manner

**DOI:** 10.1186/s12882-024-03470-9

**Published:** 2024-01-23

**Authors:** Rebecca Lehmann, Oliver Ritter, Johanna Tennigkeit, Susann Patschan, Daniel Patschan

**Affiliations:** 1 Department of Internal Medicine I - Cardiology, Nephrology and Internal Intensive Care Medicine Brandenburg University Hospital, Brandenburg Medical School (Theodor Fontane), Hochstraße 29, 14770 Brandenburg an der Havel, Germany; 2 Faculty of Health Sciences (FGW), joint faculty of the University of Potsdam, the Brandenburg Medical School Theodor Fontane and the Brandenburg Technical University Cottbus-Senftenberg, Brandenburg an der Havel, Germany

**Keywords:** AKI, BGA, pH, pCO_2_, Actual bicarbonate, Survival, Dialysis, Recovery of kidney function

## Abstract

**Background and aim:**

Acute kidney injury (AKI) is becoming increasingly prevalent among hospitalized patients and carries a poor prognosis. While new biomarkers show promise in identifying early stages of AKI, accurately predicting severe outcomes such as the need for kidney replacement therapy (KRT) or death remains a challenge. However, blood gas analyses (BGA) can be used to diagnose life-threatening complications associated with AKI. The objective of this study was to assess the role of BGA as a biomarker panel in both emerging and established cases of AKI.

**Methods:**

Retrospective observational study examining subjects with newly developed acute kidney injury (AKI). The study will document venous and arterial pH, pCO2, and actual bicarbonate levels upon hospital admission and at the onset of AKI. The primary endpoints include in-hospital mortality, the need for kidney replacement therapy (KRT), and the recovery of kidney function (ROKF).

**Results:**

A total of 202 individuals were included in the study. Three variables were found to be independent predictors of in-hospital survival: admission arterial pH, arterial pH at acute kidney injury (AKI) onset, and arterial pCO2 at AKI onset. Additionally, venous pCO2 at AKI onset was identified as an independent predictor for the need of kidney replacement therapy (KRT).

**Conclusions:**

Our study suggests that blood gas analysis may have a potential role in predicting severe outcome variables in acute kidney injury (AKI). The associated costs are minimal.

## Introduction

From a global perspective, acute kidney injury (AKI) has a higher cumulative mortality rate than heart failure, diabetes mellitus, and breast and prostate cancer combined [1]. The diagnosis of AKI is currently based on the 2012 KDIGO criteria [2], which consider changes in serum creatinine and/or urine output. Although several biomarkers have been identified, none of them or their combinations can reliably replace serum creatinine. In 2020, Ostermann et al. [3]. published "Recommendations on Acute Kidney Injury Biomarkers," a consensus statement from the Acute Disease Quality Initiative Consensus Conference. This article discussed the potential role of specific stress/damage/functional biomarkers in five categories: risk assessment, prediction of AKI, diagnosis of AKI, severity of AKI, and kidney recovery. However, two categories were missing: kidney replacement therapy (KRT) and in-hospital survival. Nonetheless, selected damage biomarkers are likely to be included in future AKI definition criteria [4], although the final decision on specific candidates is still pending.

Blood gas analyses (BGA) are routinely performed in hospitals worldwide, with negligible costs. They provide valuable information about pulmonary gas exchange and extracellular levels of hydrogen ions, sodium, potassium, and other electrolytes/metabolites. In AKI, BGA can help identify individuals at higher risk for KRT (e.g., those with progressive hyperkalemia and metabolic acidosis).

However, only a few studies have evaluated the role of BGA as an AKI "biomarker panel". In 2017, Hu and colleagues [5] conducted a retrospective study involving adult non-respiratory patients. They screened a total of 71,089 subjects admitted to the hospital between October 2014 and September 2015 (1 year), and surprisingly, only around 4,900 received BGA. The results of arterial blood gas analysis showed associations between acidosis in general, metabolic acidosis, lower actual bicarbonate levels, and hypocapnia at admission with the onset of AKI during in-hospital treatment. Additionally, hypocapnia and acidosis in general were independent predictors of in-hospital death. Lower venous pCO2 at admission was also independently predictive of both AKI onset and in-hospital death. The authors also found predictive associations between certain electrolyte imbalances and AKI onset (hypo- and hypernatremia, hypochloremia, hypocalcemia, hypomagnesemia, hypo- and hyperphosphatemia). However, they did not draw mechanistic conclusions regarding the relationship between BGA abnormalities and outcome variables, as these abnormalities may have reflected the severity of the underlying disorder, which could have contributed to a higher risk profile.

This current investigation aimed to evaluate the predictive role of three BGA variables (pH, actual bicarbonate, and pCO2) in individuals with de novo AKI. BGA results from two time points were considered: hospital admission and the appearance of clinical AKI (AKI onset). Three endpoints were defined: in-hospital death, the need for KRT, and recovery of kidney function (ROKF) until discharge.

## Methods

### Design

The investigation was conducted as a single-center, retrospective, observational study. All patients were recruited from the University Hospital of Brandenburg, affiliated with the Medical School of Brandenburg. The study was formally approved by the medical school's ethics committee (E-01–20210510). Written consent from the participants was not required due to the retrospective design. The recruitment period spanned from January to December 2019. Potentially eligible subjects were identified using the hospital's electronic acute kidney injury (AKI) alert system, which is based on criteria 1 and 2 of the 2012 published "KDIGO clinical practice guidelines for acute kidney injury" [2]. If at least one criterion was met, an automated message containing anonymized patient information was generated and sent to the responsible nephrologist. All relevant medical information pertaining to the cases was extracted from the hospital's central database (MEDICO®, CompuGroupMedical – CGM), which also included all laboratory findings obtained during each patient's in-hospital stay.

### Endpoints

The primary objective of the study was to assess in-hospital mortality. Secondary objectives included evaluating the need for kidney replacement therapy (KRT) and the recovery of kidney function (ROKF). The criterion for determining the need for KRT was met if at least one session of extracorporeal treatment was required. KRT could be administered as intermittent hemodialysis, hemodiafiltration, or slow extended daily dialysis (SLEDD). Generally, KRT was initiated if any of the following criteria were met: persistent fluid overload with oligo-anuria accompanied by resistant hypertension, pulmonary congestion, and the need for non-invasive or invasive ventilation; refractory hyperkalemia (serum potassium > 6.5 mmol/l) despite the use of at least two of the following medications – insulin + glucose, intravenous bicarbonate, loop diuretics; refractory acidemia (arterial pH < 7.15) despite the use of intravenous bicarbonate; and presence of uremia-related symptoms (unexplained neurological symptoms, pruritus, nausea and vomiting, loss of appetite). Ultimately, the decision to initiate KRT was made by the responsible nephrologist. ROKF was categorized as complete or incomplete. Complete ROKF was diagnosed if the last estimated glomerular filtration rate (eGFR) differed from the highest eGFR by no more than 10 mL/min, while incomplete ROKF was diagnosed if the difference was greater than 10 but not exceeding 20 mL/min. The decision to use eGFR for ROKF assessment was based on the concept of Acute Kidney Disease, which considers a persistent decrease in eGFR over time.

### Statistics

Comparisons between two groups were conducted using the Chi-square test for categorical data. The normal distribution of numerical data was initially assessed using the Kolmogorov–Smirnov test. If the data followed a normal distribution, comparisons between two groups were performed using the Student's t-test. For non-normally distributed data, the Mann–Whitney test was used for comparisons. Comparisons involving more than two groups were analyzed using either ANOVA (for normally distributed data) or the Kruskal–Wallis test (for non-normally distributed data). Statistical significance was defined as a p-value below 0.05. Results were reported as percentages, mean ± standard deviation, or median ± interquartile range. Multivariate logistic regression analysis was performed to assess the association between eight covariates (including one blood gas analysis parameter, gender, SOFA score, coronary artery disease, heart insufficiency, chronic kidney disease, obesity, and diabetes) and either in-hospital survival or the need for kidney replacement therapy (KRT). In this analysis, in-hospital survival was considered the primary endpoint, while the need for KRT was the secondary endpoint. All statistical analyses were conducted using WIZARD® for MacOS (version 2.0.11, developed by Evan Miller).

## Results

### Baseline characteristics

During the observational period, a total of 202 individuals were included in the study. Of these, 132 (65.3%) were females and 70 (34.7%) were males. The mean age of all individuals was 74.1 ± 13.6 years. The average in-hospital treatment time was 19.2 ± 15.7 days.

According to the KDIGO classification [2] the distribution of AKI stages among the participants was as follows: stage 1—23.3%, stage 2—17.3%, and stage 3—59.4%. The overall in-hospital survival rate was 65.8%, patients that required ICU therapy (52%) died in 78.3%. Kidney replacement therapy (KRT) was required in 20.8% of the cases. Complete kidney function recovery (ROKF) was observed in 39.7% of the patients, while incomplete recovery was seen in 29.3%.

The main reasons for hospitalization among the participants were as follows: heart failure (15.3%), sepsis (9.9%), general weakness (7.9%), non-septic infection (7.9%), acute abdomen (6.9%), bleeding with or without shock (6.9%), dyspnea of unknown origin (6.9%), syncope (6.9%), dehydration (5%), and other (26.2%).

The baseline characteristics of the patients are summarized in Table 1. Table 2 lists the etiology of AKI.

**Table 1:** Baseline characteristics of all patients included

Variable	Result
General characteristics	
Gender (females / males)	*n* = 70 and 34.7% / *n* = 132 and 65.3%
Age (years ± SD)	74.1 ± 13.6
In-hospital therapy (days ± SD)	19.2 ± 15.7
AKI stage according to KDIGO (I / II / II)	23.3% / 17.3% / 59.4%
SOFA score on admission	3.42 ± 2.8
In-hospital survival all individuals	65.8%
In-hospital survival ICU treated patients	21.7%
KRT all individuals	20.8%
KRT ICU treated individuals	23.7%
ROKF (complete / incomplete / no recovery)	39.7% / 29.3% / 31%
Morbidities	
Arterial hypertension	78.7%
Diabetes mellitus	36.6%
Obesity	29.7%
Hyperuricemia	7.9%
Coronary artery disease	41.6%
Chronic heart failure	34.2%
Chronic kidney disease	34.8%
COPD	19.8%
History of neoplasia	21.8%
Smoking	17.8%
ICU therapy / invasive measures	
ICU therapy	52%
Vasopressors	46%
Ventilatory therapy	45%
Blood gas analyzes	
Admission venous pH	7.32 ± 0.16
Admission venous pCO_2_ (mmHg)	38.9 ± 10.9
Admission venous bicarbonate (mMol/L)	20.42 ± 7.1
Venous pH at AKI onset	7.29 ± 0.17
Venous pCO_2_ at AKI onset (mmHg)	39.1 ± 10.8
Venous bicarbonate at AKI onset (mMol/L)	19.8 ± 7.3
Admission arterial pH	7.31 ± 0.16
Admission arterial pCO_2_ (mmHg)	38.7 ± 12.7
Admission arterial bicarbonate (mMol/L)	19.8 ± 6.1
Arterial pH at AKI onset	7.3 ± 0.15
Arterial pCO_2_ at AKI onset (mmHg)	41.6 ± 13.1
Arterial bicarbonate at AKI onset (mMol/L)	20.3 ± 6.6

**Table 2:** Etiology of AKI

AKI etiology	%
Pre-renal	27.7
Sepsis	25.2
Cardiorenal	19.3
Drug-induced	2
Obstruction	1
Other	9.4
Unknown	15.4

### Blood gas analysis data sets

The data sets for blood gas analysis were incomplete. Complete sets were available for the following percentages: 43.1% for admission venous pH / pCO2 / bicarbonate, 35.6% for venous pH / pCO2 / bicarbonate at AKI onset, 37.6% for admission arterial pH / pCO2 / bicarbonate, and 54.5% for arterial pH / pCO2 / bicarbonate at AKI onset. Table 1 shows the means ± standard deviation, and Table 3 summarizes the variables in patients who reached the pre-defined endpoints, rather than including subjects who did not.

**Table 3:** all BGA variables in patients reaching the pre-defined endpoints as opposed to subjects that did not

BGA variable	Endpoint				*p*-value	
	survival		death			
Admission venous pH	7.34 ± 0.01		7.27 ± 0.04		0.4	
Admission venous pCO_2_ (mmHg)	39.7 ± 1.2		38.5 ± 2.3		0.49	
Admission venous bicarbonate (mMol/L)	21.4 ± 0.8		18.8 ± 1.5		0.24	
Venous pH at AKI onset	7.33 ± 0.01		7.2 ± 0.05		**0.037**	
Venous pCO_2_ at AKI onset (mmHg)	39.4 ± 1.4		38.7 ± 2.9		0.8	
Venous bicarbonate at AKI onset (mMol/L)	21.2 ± 0.9		16.3 ± 1.6		**0.04**	
Admission arterial pH	7.36 ± 0.01		7.22 ± 0.04		**0.003**	
Admission arterial pCO_2_ (mmHg)	35.5 ± 1.2		44.8 ± 3.1		**0.03**	
Admission arterial bicarbonate (mMol/L)	20.2 ± 0.8		19.1 ± 1.3		0.64	
Arterial pH at AKI onset	7.34 ± 0.01		7.24 ± 0.02		**0.002**	
Arterial pCO_2_ at AKI onset (mmHg)	37.6 ± 1.1		46.8 ± 2.3		** < 0.001**	
Arterial bicarbonate at AKI onset (mMol/L)	20.2 ± 0.6		20.4 ± 1.1		0.64	
	no KRT		KRT			
Admission venous pH	7.34 ± 0.01		7.24 ± 0.05		0.25	
Admission venous pCO_2_ (mmHg)	39.9 ± 1.3		35.9 ± 2.2		0.16	
Admission venous bicarbonate (mMol/L)	21.7 ± 0.7		17.3 ± 1.7		**0.049**	
Venous pH at AKI onset	7.31 ± 0.02		7.25 ± 0.05		0.64	
Venous pCO_2_ at AKI onset (mmHg)	41.2 ± 1.4		34.2 ± 2.1		**0.02**	
Venous bicarbonate at AKI onset (mMol/L)	20.8 ± 0.9		17.1 ± 1.7		0.12	
Admission arterial pH	7.33 ± 0.01		7.26 ± 0.05		0.18	
Admission arterial pCO_2_ (mmHg)	38.7 ± 1.6		38.4 ± 3.5		0.72	
Admission arterial bicarbonate (mMol/L)	20.2 ± 0.7		18.4 ± 1.9		0.7	
Arterial pH at AKI onset	7.31 ± 0.01		7.26 ± 0.03		0.08	
Arterial pCO_2_ at AKI onset (mmHg)	41.8 ± 1.4		41 ± 2.6		0.85	
Arterial bicarbonate at AKI onset (mMol/L)	20.7 ± 0.6		19 ± 1.5		0.35	
	complete ROKF	incomplete ROKF		no ROKF		
Admission venous pH	7.34 ± 0.03	7.3 ± 0.01		7.37 ± 0.02		0.05
Admission venous pCO_2_ (mmHg)	41.7 ± 2.2	39.6 ± 1.9		37.6 ± 2.8		0.44
Admission venous bicarbonate (mMol/L)	22.5 ± 1.5	19.5 ± 1.1		21.3 ± 1.8		0.33
Venous pH at AKI onset	7.38 ± 0.02	7.25 ± 0.02		7.34 ± 0.03		0.004
Venous pCO_2_ at AKI onset (mmHg)	39.2 ± 2.1	39.4 ± 2.7		39.3 ± 2.3		0.95
Venous bicarbonate at AKI onset (mMol/L)	23 ± 1.3	17.7 ± 1.4		21.9 ± 1.8		0.07
Admission arterial pH	7.36 ± 0.02	7.33 ± 0.03		7.38 ± 0.01		0.55
Admission arterial pCO_2_ (mmHg)	37.6 ± 1.9	33.9 ± 3.4		32.6 ± 1.2		0.33
Admission arterial bicarbonate (mMol/L)	21.4 ± 1.3	18.1 ± 1.9		19.4 ± 1		0.32
Arterial pH at AKI onset	7.32 ± 0.02	7.35 ± 0.02		7.35 ± 0.01		0.56
Arterial pCO_2_ at AKI onset (mmHg)	40.4 ± 1.7	33.8 ± 1.8		36.9 ± 2		0.12
Arterial bicarbonate at AKI onset (mMol/L)	20.8 ± 1	17.9 ± 1.4		20.3 ± 1.2		0.34

### Survival

**Fig. 1:**
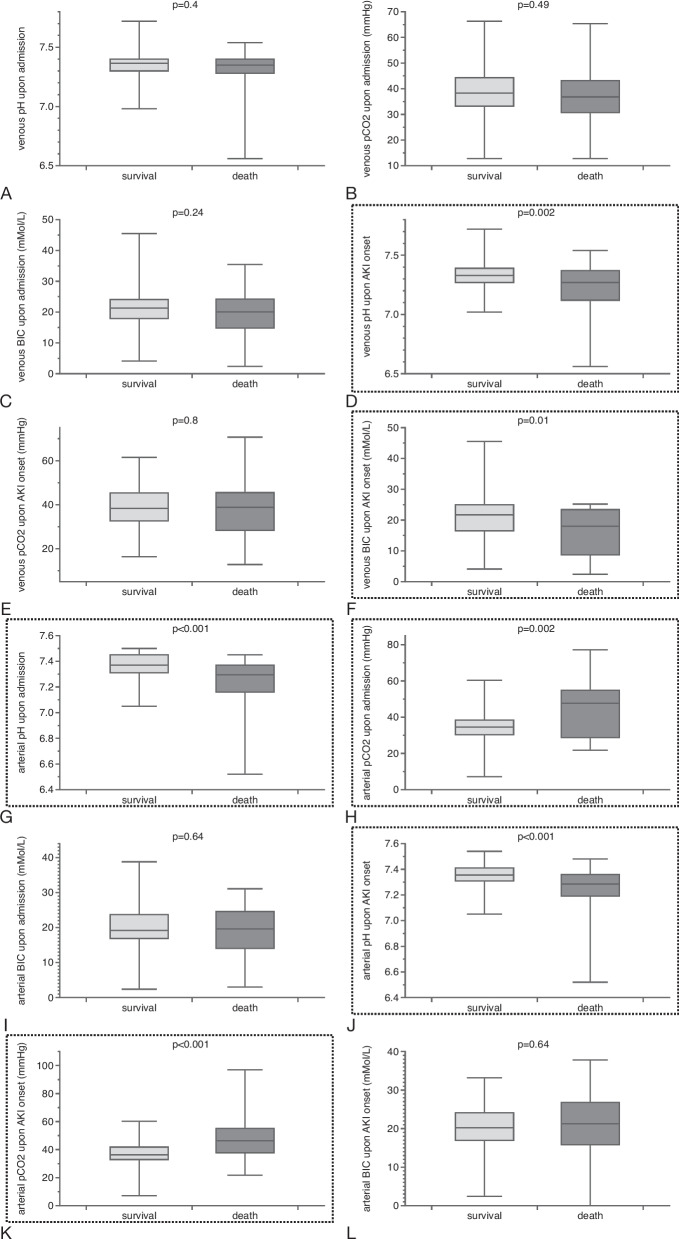
Results of all venous and arterial blood gas analyses, including pH, pCO2, and current bicarbonate levels, in relation to the survival endpoint. Significant differences were observed for the following variables: venous pH at the time of clinical AKI diagnosis (**D**), venous bicarbonate at the time of AKI diagnosis (**F**), arterial pH at initial presentation (**G**), arterial pCO2 at presentation (**H**), as well as arterial pH and pCO2 at the time of AKI diagnosis (**J** and **K**) (the dashed rectangles indicate comparisons with statistical significance; results as median ± IQR)

The blood gas analysis (BGA) revealed significant differences between survivors and non-survivors in various variables. Non-survivors had lower venous pH and bicarbonate levels at AKI onset, as well as lower arterial pH at admission. On the other hand, non-survivors had higher arterial pCO2 at admission. Conversely, survivors had higher arterial pH at AKI onset and lower arterial pCO2 at AKI onset (Table 3 and Fig. 1).

Univariate analysis confirmed that all six variables were significantly associated with survival (Table 4). These variables were then included in a multivariate logistic analysis, along with other covariates such as gender, SOFA score, coronary artery disease, heart insufficiency, chronic kidney disease, obesity, and diabetes. Three variables—admission arterial pH, arterial pH at AKI onset, and arterial pCO2 at AKI onset—were identified as independent predictors of in-hospital survival.

**Table 4:** Univariate analyses of all blood gas variables which differed in certain endpoint catego-ries (survival, KRT – kidney replacement therapy, ROKF – recovery of kidney function). All variables but venous pH at AKI onset (endpoint ROKF) entered multivariate analysis

Variable	Endpoint	AUC	*p*-value
Venous pH at AKI onset	survival	0.65	**0.01**
Venous pH at AKI onset	ROKF	0.54	0.53
Venous pCO_2_ at AKI onset	KRT	0.67	**0.01**
Venous bicarbonate at AKI onset	survival	0.65	**0.01**
Admission arterial pH	survival	0.71	**0.004**
Arterial pH at AKI onset	survival	0.67	**0.003**
Admission arterial pCO_2_	survival	0.65	**0.005**
Arterial pCO_2_ at AKI onset	survival	0.69	** < 0.001**

### KRT

**Fig. 2:**
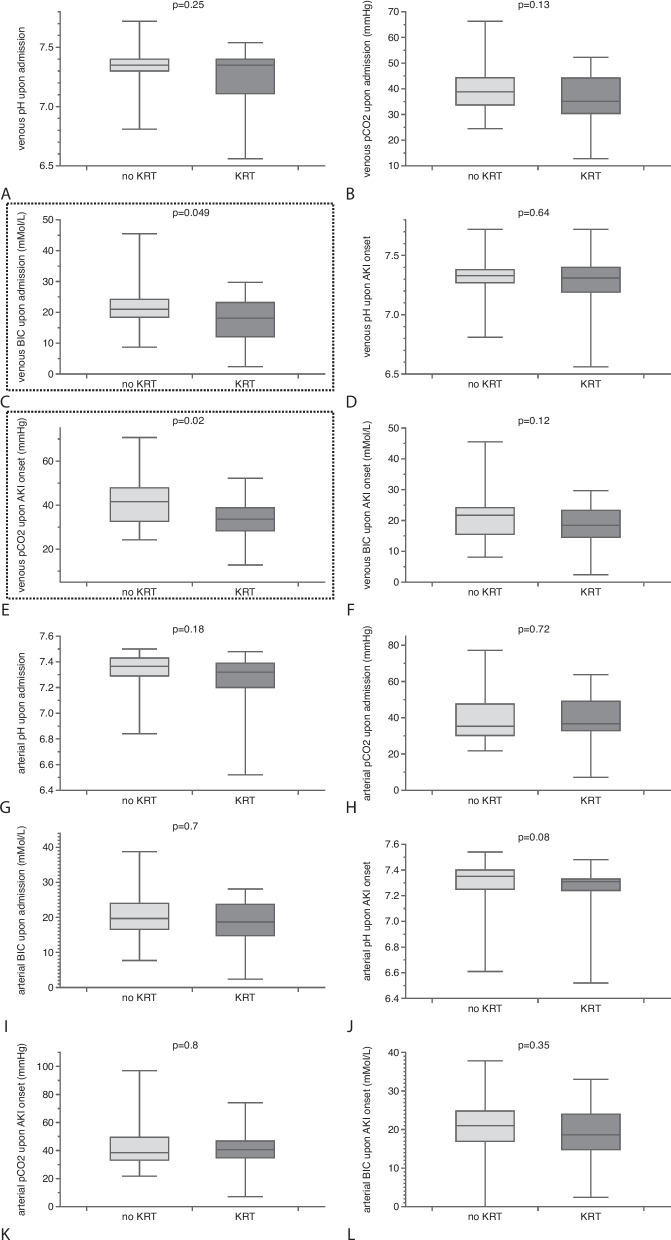
Blood gas analyses (venous and arterial pH, pCO2, and current bicarbonate concentration) in relation to the endpoint of renal replacement therapy. Two variables differed significantly between individuals with and without dialysis: venous bicarbonate concentration at admission (C) and venous pCO2 at AKI diagnosis (E) (the dashed rectangles indicate comparisons with statistical significance; results as median ± IQR)

We identified two differences between individuals who required kidney replacement therapy (KRT) and those who did not: venous pCO2 levels at the onset of acute kidney injury (AKI) and admission venous bicarbonate were higher in patients who did not require dialysis (Table 3 and Fig. 2). Since the variable venous bicarbonate concentration barely reached the statistical significance level upon admission, it was not considered in further analyses. Venous pCO2 at AKI onset was found to be independently predictive for the need for KRT, as confirmed by both univariate and multivariate analysis (Tables 4 and 5).

**Table 5:** Multivariate logistic regression analysis. In-hospital survival was defined as dependent variable (primary endpoint), 7 covariables entered the analyses: one BGA covariable (first column), gender, SOFA score, coronary artery disease, heart insufficiency, chronic kidney disease, obesity, and diabetes. One BGA covariable (venous pCO_2_ at AKI onset) was independently predictive regarding the dependent variable KRT (secondary endpoint)

BGA covariable	Endpoint	*p*-value
Venous pH at AKI onset	survival	0.24
Venous pCO_2_ at AKI onset	KRT	**0.006**
Venous bicarbonate at AKI onset	survival	0.463
Admission arterial pH	survival	**0.019**
Arterial pH at AKI onset	survival	**0.003**
Admission arterial pCO_2_	survival	0.007
Arterial pCO_2_ at AKI onset	survival	** < 0.001**

### ROKF

The venous pH at the onset of acute kidney injury (AKI) showed significant differences between the categories of "complete recovery," "incomplete recovery," and "no recovery" (*p* < 0.001) (Table 3). Further subgroup analysis indicated significant differences between the "complete recovery" category compared to the other two categories (*p* = 0.015), as well as between the "incomplete recovery" category and the other two categories (*p* = 0.001).

## Discussion

At this point, it briefly needs to be reiterated why the study aimed to evaluate the value of blood gas analysis emergin or newly established AKI. Predicting relevant clinical outcomes (such as the need for dialysis or death) in AKI remains challenging. Blood gas tests are cost-effective and widely available. The inclusion of both venous and arterial analyses in the current pilot study was based on the limited existing data on this topic.

We identified three independent predictors of in-hospital survival in subjects with AKI: admission arterial pH, arterial pH at AKI onset, and arterial pCO2 at AKI onset. Additionally, venous pCO2 at AKI onset was found to be predictive of the need for KRT. These findings are particularly intriguing considering that blood gas analysis data was not available for all subjects, suggesting the potential role of these variables as prognostic biomarkers for emerging AKI, even before clinical diagnosis.

A retrospective study by Hu et al. [5] - excuse me: the dots behind all reference numbers are placed there according to the journal style ? I am not sure in 2017 also demonstrated that acidosis in general, metabolic acidosis, lower actual bicarbonate, and hypocapnia at the time of hospital admission were predictive of both hospital-acquired AKI and in-hospital mortality. Furthermore, associations were found between hypo- and hypernatremia, hypochloremia, hypocalcemia, hypomagnesemia, and hypo- and hyperphosphatemia, respectively.

An ideal biomarker for AKI would provide diagnostic and prognostic information as early as possible. Nickolas et al. [6] collected urine samples from more than 1,600 patients at the emergency department and quantified the following biomarkers: NGAL, KIM-1, L-FABP, IL-18, and cystatin C. Both urine NGAL and KIM-1 significantly enhanced the predictive power of a creatinine-based model for in-hospital mortality and the need for KRT. In other words, these markers helped identify AKI patients at risk of death or requiring KRT even before the diagnosis of AKI was made. In a study published in 2021, Gisewhite et al. [7] conducted a secondary analysis of urine samples from 82 patients with combat-associated injuries [8]. The study aimed to analyze metabolites in the urine and their association with death, the need for kidney replacement therapy (KRT), and the severity of acute kidney injury (AKI). The authors employed a metabolomics approach [9, 10] and used proton nuclear magnetic resonance (1H-NMR) spectroscopy for all the analyses. They identified nine urine metabolites (lactate, glucose, 1-methylnicotinamide, 2-hydroxybutyrate, glycine, pyruvate, 2-hydroxyvalerate, 1,6-anhydro-beta-D-glucose, threonine) that were associated with death and the need for KRT during follow-up. Additionally, eleven metabolites (1-methylnicotinamide, lactate, glycine, citrate, 3-hydroxyisovalerate, hippurate, histidine, xanthosine, 3-indoxylsulfate, tartrate, threonine, phenylacetylglycine, 1,6-anhydro-beta-D-glucose, glucose, pyruvate, indole-3-acetate) were associated with the severity of AKI. Of particular interest, increased levels of 1-methylnicotinamide were predictive of mortality, the need for KRT, and higher stages of AKI. Conversely, increased levels of glycine were indicative of survival, no need for KRT, and less severe AKI.

The "Recommendations on Acute Kidney Injury Biomarkers" [3] categorized the clinical application of biomarkers into five categories: risk assessment, prediction of AKI, diagnosis of AKI, severity of AKI, and kidney recovery. While most biomarkers provided diagnostic information, only three were identified for kidney recovery (C–C motif chemokine ligand 14 [11], Hepatocyte Growth Factor [12], proenkephalin A [13]. The category of the need for kidney replacement therapy (KRT) was not considered at all. However, from a clinical perspective, early identification of patients at risk for KRT is crucial. The independent association between venous pCO2 at AKI onset and the need for KRT during follow-up lacks however a reasonable explanation at the moment.

By searching for the terms "pCO2" and "AKI," a total of 8 references were found as of September 2022. One study examined the relationship between oxygen delivery (DO2), carbon dioxide production (VCO2), and the incidence of acute kidney injury (AKI) following cardiopulmonary bypass surgery [14]. The study found that a decrease in DO2 was associated with AKI, while VCO2 did not show a significant association. It is worth noting that both DO2 and VCO2 are not commonly used blood gas analysis variables. However, there is a lack of additional references specifically discussing the use of pCO2 as a biomarker for AKI.

There are limitations of our study that need to be mentioned. Firstly, the study design was retrospective, which may introduce bias. Secondly, there were incomplete blood gas analysis data sets, limiting the analysis. This is surprising considering the immediate diagnostic benefits of blood gas analysis in identifying complications associated with acute kidney injury (AKI) such as hyperkalemia and metabolic acidosis. However, even with these limited data, promising results have been identified. The data justifies, in our opinion, a follow-up study with a prospective, multicenter design. Thirdly, information on urine output was lacking for many individuals, preventing the consideration of criterion 3 of the KDIGO guideline. The often neglected measurement of urine volume may also reflect a latent lack of physician awareness regarding the topic of AKI, as discussed in the studies by Ali et al. [15] and Adejumo et al. [16]. Overall, this may have resulted in the missed identification of some patients with early AKI. Additionally, there was heterogeneity in the admission baseline characteristics, with patients requiring KRT showing higher SOFA scores, indicating a higher degree of morbidity from the beginning. Therefore, BGA abnormalities may simply reflect the degree of morbidity rather than being directly associated with AKI.

In summary, our study nevertheless suggests a potential role for blood gas analysis in predicting the morbidity and mortality risk in AKI. The costs are minimal, and several variables were found to be independently predictive. Even venous variables measured at AKI onset were able to predict in-hospital death and the need for KRT. These findings highlight the importance of blood gas analysis in the management of emerging or established AKI. However, there is also a need to improve physicians' awareness and knowledge of AKI [15, 16] to ensure the appropriate utilization of blood gas analysis. A larger prospective trial is necessary to confirm these findings, and future trials should aim to homogenize baseline characteristics upon admission.

## Data Availability

The data supporting the findings of this study are available from the corresponding author upon reasonable request.: d.patschan@klinikum-brandenburg.
